# The next evolution of theranostics: from target discovery to integrated precision medicine

**DOI:** 10.3389/fmed.2026.1937715

**Published:** 2026-08-18

**Authors:** Christopher Montemagno, Benjamin Serrano, Benoit Paulmier

**Affiliations:** 1 Medical Biology Department, Centre Scientifique de Monaco, Monaco, Monaco; 2 Medical Physics Department, Princess Grace Hospital, Monaco, Monaco; 3 Department of Nuclear Medicine, Princess Grace Hospital, Monaco, Monaco

**Keywords:** molecular imaging, molecular targets, personalized dosimetry, precision medicine, radiopharmaceutical therapy (RPT), theranostics

## 1 Introduction

For more than two decades, innovation in theranostics has been driven by one central scientific question: which molecular target should be imaged and treated next? This quest for actionable molecular targets transformed nuclear medicine, leading to landmark advances such as peptide receptor radionuclide therapy (PRRT) for neuroendocrine tumors and prostate-specific membrane antigen (PSMA)-targeted radioligand therapy (RLT) for metastatic castration-resistant prostate cancer. The NETTER-1 and VISION phase III trials, together with subsequent EANM/SNMMI guidelines, established theranostics as one of the most successful examples of precision oncology (1–4).

Today, however, the field may be approaching a different challenge. As the number of clinically actionable molecular targets continues to expand, discovering another target is no longer sufficient, by itself, to drive the next major advances in theranostics. Increasingly, clinical impact depends on how effectively existing and emerging targets can be integrated into a broader precision medicine strategy.

This evolution is reflected across the theranostic pathway. Quantitative molecular imaging, patient stratification, individualized dosimetry, computational treatment planning, therapeutic optimization, longitudinal response assessment, and adaptive treatment strategies are becoming major determinants of patient benefit rather than complementary components of radionuclide therapy (5–9). Collectively, these developments share a common objective: maximizing the clinical value of molecular targets that have already been identified.

**Figure 1:**
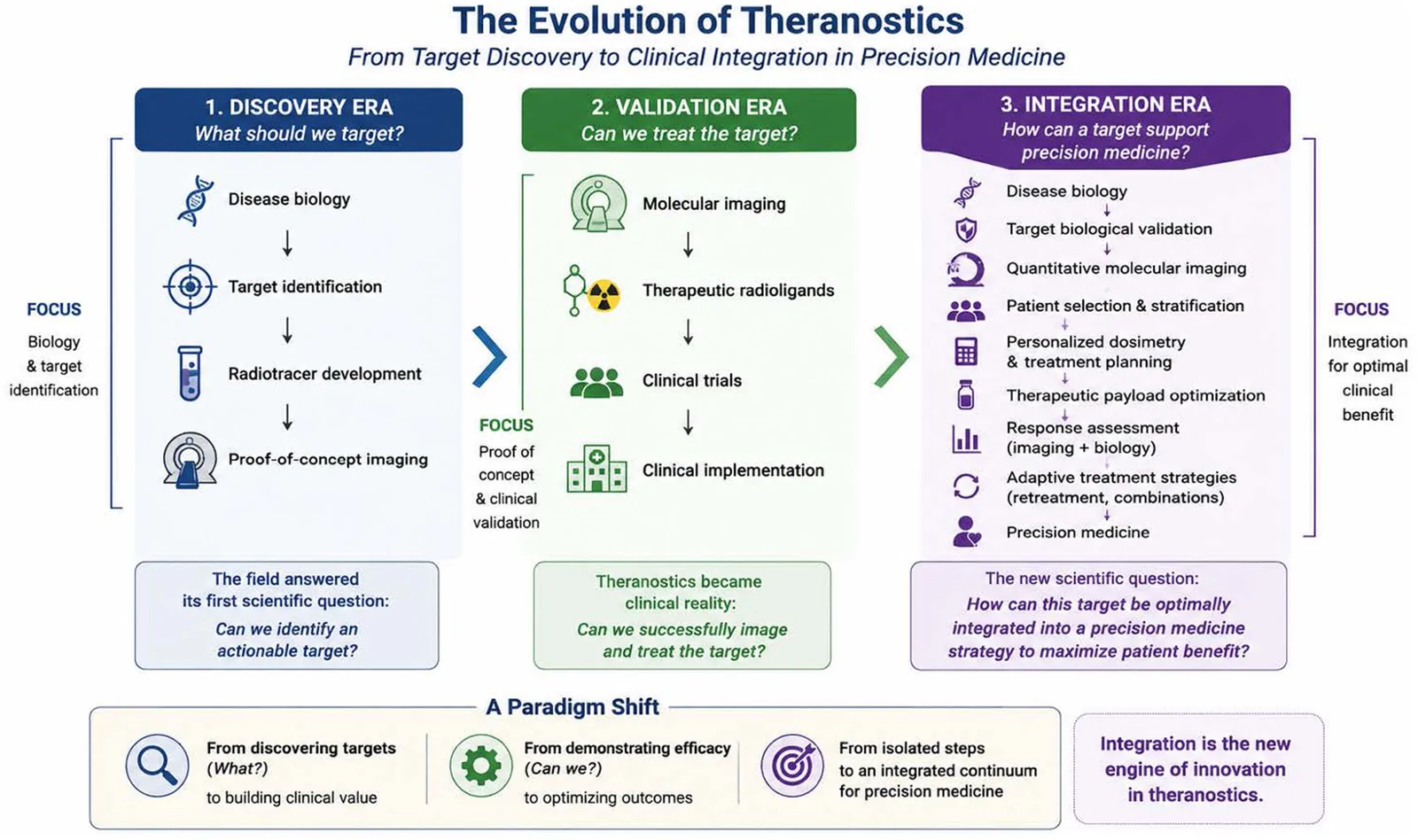
Conceptual framework illustrating the evolution of theranostics. The Discovery Era focused on identifying actionable molecular targets and developing target-specific radiopharmaceuticals. The Validation Era established the clinical utility of these targets for molecular imaging and radionuclide therapy. The proposed Integration Era emphasizes the incorporation of molecular targets into comprehensive precision medicine workflows, including quantitative imaging, patient selection, personalized dosimetry, therapeutic optimization, response assessment, and adaptive treatment strategies.

This Opinion argues that theranostics is entering a new stage of development. Future breakthroughs will undoubtedly continue to emerge from novel biological targets, but the principal driver of innovation is progressively shifting from target discovery toward the integrated clinical exploitation of those targets. This transition—from target discovery to integrated precision medicine—forms the conceptual basis of this Opinion. As illustrated in Figure 1, we propose that theranostics is entering an Integration Era in which the clinical value of molecular targets increasingly depends on their incorporation into comprehensive precision medicine workflows rather than on target identification alone.

## 2 Target discovery transformed theranostics

The success of theranostics was built upon a deceptively simple concept: a single molecular target could serve as a common biological gateway for both diagnosis and therapy. By linking molecular imaging with targeted radionuclide therapy, this principle fundamentally redefined the role of nuclear medicine, transforming it from a predominantly diagnostic specialty into a discipline capable of guiding and delivering personalized treatment. The demonstration that one molecular target could support disease visualization, patient selection, and therapeutic intervention represented a conceptual breakthrough that distinguished theranostics from both conventional imaging and systemic therapies (1, 2).

These achievements naturally stimulated an intense search for additional theranostic targets. Fibroblast activation protein (FAP), CXCR4, gastrin-releasing peptide receptor (GRPR), B7-H3, integrins, and numerous other emerging biomarkers continue to broaden the theranostic landscape, creating new opportunities across oncology and beyond (10–12). This continued diversification remains a major driver of innovation and will undoubtedly expand the clinical applications of radiopharmaceutical sciences.

Importantly, this expansion should not be interpreted as a competition to identify the next major theranostic breakthrough. Rather, each new molecular target broadens the theranostic paradigm by addressing distinct tumor biologies, disease processes, and clinical settings.

At the same time, however, the success of this target-driven innovation has fundamentally changed the questions facing the field. As the number of actionable molecular targets continues to increase, discovering another target is no longer sufficient, by itself, to ensure meaningful clinical impact. Instead, the challenge is progressively shifting toward understanding how each target can be translated into the greatest possible patient benefit. The central scientific question is therefore evolving from “What can we target?” to “How can we best use the targets we already have?”

## 3 The bottleneck has shifted

The success of theranostics has not eliminated the need for new molecular targets; it has changed the factors that determine their clinical impact. While target discovery remains fundamental, it is no longer sufficient, by itself, to maximize patient benefit. Increasingly, innovation depends on how effectively molecular targets are translated into clinical practice and integrated into precision medicine workflows.

This shift is evident across virtually every stage of the theranostic pathway. Quantitative molecular imaging is evolving beyond lesion detection toward treatment planning and response prediction. Personalized dosimetry is increasingly recognized as an essential component of radiopharmaceutical therapy and is progressively moving beyond fixed-activity approaches to account for the substantial variability in radiation dose delivered to individual patients. Similarly, advances in computational modeling, artificial intelligence, treatment sequencing, and combination strategies all pursue a common objective: extracting greater clinical value from existing molecular targets rather than simply expanding the catalog of actionable biomarkers (5–8).

These developments highlight an important conceptual distinction. Identifying a biologically relevant target is only the first step toward successful theranostics. Clinical benefit increasingly depends on a much broader set of factors, including robust patient selection, quantitative imaging, optimized radionuclide delivery, individualized dosimetry, response assessment, and treatment adaptation (5, 7, 9). Consequently, the clinical value of a molecular target can no longer be inferred solely from its biological specificity or expression profile.

Personalized dosimetry illustrates this evolution particularly well. Historically, radiopharmaceutical therapies have generally been administered using standardized activity regimens. However, considerable interpatient variability in absorbed dose has demonstrated that identical administered activities rarely produce equivalent biological effects. Recent international recommendations increasingly advocate quantitative post-treatment imaging and patient-specific dosimetric approaches, reflecting a growing consensus that individualized dosimetry has the potential to optimize radiopharmaceutical therapy (5, 7). However, despite compelling evidence supporting its clinical value, widespread implementation in routine clinical practice remains limited outside selected indications such as selective internal radiotherapy (SIRT) (13, 14).

This evolution also changes how future theranostic targets should be evaluated. The critical question is no longer simply whether a novel molecular target can support both imaging and therapy, but whether it can be effectively integrated into a clinical strategy capable of improving patient outcomes. Future innovation will therefore depend not only on discovering new molecular targets, but also on maximizing the clinical value of existing and emerging ones.

## 4 Redefining the evaluation of future theranostic targets

If the principal driver of innovation in theranostics is progressively shifting from target discovery to clinical integration, the criteria used to evaluate emerging molecular targets must also evolve. Historically, new theranostic targets were primarily assessed according to their biological specificity and their ability to support both imaging and therapy. Although these characteristics remain essential, they are no longer sufficient to predict clinical success.

Future theranostic targets should increasingly be evaluated according to the clinical value they can generate within an integrated precision medicine strategy. Beyond target expression alone, clinically relevant questions include whether quantitative imaging can reliably guide patient selection, whether individualized dosimetry can optimize treatment delivery, whether different radionuclides or therapeutic payloads can be exploited, whether treatment response can be monitored longitudinally, and whether the target can be incorporated into adaptive therapeutic strategies or rational combination therapies (5–9). Importantly, this broader evaluation is particularly relevant for targets predominantly expressed within the tumor microenvironment or peritumoral tissues, where excellent diagnostic performance does not necessarily translate into therapeutic efficacy. Clinical experience with FAP-targeted ligands and PSMA-directed radioligand therapy in selected non-prostate malignancies further illustrates that biological target expression alone may not be sufficient to predict therapeutic success.

This broader perspective also explains why emerging molecular targets follow markedly different developmental trajectories despite compelling biological rationale. Some rapidly advance toward routine clinical implementation, whereas others demonstrate excellent imaging characteristics but more limited therapeutic efficacy. Such differences may reflect not only target biology, but also the ability of a target to support reproducible imaging protocols, standardized patient selection, individualized treatment planning, and robust therapeutic algorithms.

Emerging biomarkers such as FAP, CXCR4, integrins, and B7-H3 illustrate this evolving landscape. Their clinical value will likely depend not only on demonstrating target expression, but also on defining the clinical context in which they provide meaningful benefit. Rather than competing to become the next PSMA, future theranostic targets may be better viewed as complementary biological platforms that address distinct biological questions, diseases, and therapeutic needs.

## 5 Concluding remarks

Theranostics has entered a period of remarkable scientific maturity. The discovery of new molecular targets will remain fundamental to expanding the scope of radiopharmaceutical medicine, but it is unlikely to remain the sole driver of future clinical progress. Future innovation will increasingly depend on how effectively molecular targets are integrated into precision medicine strategies capable of maximizing patient benefit.

This evolution does not replace the target discovery paradigm; it builds upon it. The future of theranostics will therefore depend as much on integrating molecular targets into optimized clinical workflows as on discovering new ones. Such integration requires close collaboration across disciplines and a shift toward evaluating theranostic agents according to the clinical value they generate rather than their biological characteristics alone.

Ultimately, the next major breakthrough in theranostics may not be the discovery of another molecular target. It may instead arise from rethinking the role of molecular targets—not as the endpoint of innovation, but as the starting point of integrated precision medicine.
